# Envelope Enhancement Increases Cortical Sensitivity to Interaural Envelope Delays with Acoustic and Electric Hearing

**DOI:** 10.1371/journal.pone.0104097

**Published:** 2014-08-05

**Authors:** Douglas E. H. Hartley, Amal Isaiah

**Affiliations:** 1 NIHR National Biomedical Research Unit in Hearing, Ropewalk House, Nottingham, United Kingdom; 2 Department of Otolaryngology, School of Clinical Sciences, Nottingham University, Nottingham, United Kingdom; 3 Medical Research Council Institute of Hearing Research, University Park, Nottingham, United Kingdom; 4 Department of Physiology, Anatomy & Genetics, University of Oxford, Oxford, United Kingdom; UNLV, United States of America

## Abstract

Evidence from human psychophysical and animal electrophysiological studies suggests that sensitivity to interaural time delay (ITD) in the modulating envelope of a high-frequency carrier can be enhanced using half-wave rectified stimuli. Recent evidence has shown potential benefits of equivalent electrical stimuli to deaf individuals with bilateral cochlear implants (CIs). In the current study we assessed the effects of envelope shape on ITD sensitivity in the primary auditory cortex of normal-hearing ferrets, and profoundly-deaf animals with bilateral CIs. In normal-hearing animals, cortical sensitivity to ITDs (±1 ms in 0.1-ms steps) was assessed in response to dichotically-presented i) sinusoidal amplitude-modulated (SAM) and ii) half-wave rectified (HWR) tones (100-ms duration; 70 dB SPL) presented at the best-frequency of the unit over a range of modulation frequencies. In separate experiments, adult ferrets were deafened with neomycin administration and bilaterally-implanted with intra-cochlear electrode arrays. Electrically-evoked auditory brainstem responses (EABRs) were recorded in response to bipolar electrical stimulation of the apical pair of electrodes with singe biphasic current pulses (40 µs per phase) over a range of current levels to measure hearing thresholds. Subsequently, we recorded cortical sensitivity to ITDs (±800 µs in 80-µs steps) within the envelope of SAM and HWR biphasic-pulse trains (40 µs per phase; 6000 pulses per second, 100-ms duration) over a range of modulation frequencies. In normal-hearing animals, nearly a third of cortical neurons were sensitive to envelope-ITDs in response to SAM tones. In deaf animals with bilateral CI, the proportion of ITD-sensitive cortical neurons was approximately a fifth in response to SAM pulse trains. In normal-hearing and deaf animals with bilateral CI the proportion of ITD sensitive units and neural sensitivity to ITDs increased in response to HWR, compared with SAM stimuli. Consequently, novel stimulation strategies based on envelope enhancement may prove beneficial to individuals with bilateral cochlear implants.

## Introduction

A delay in the time of arrival of a sound between the ears, termed interaural time delay (ITD), contributes to our ability to localize sounds, detect speech in background noise and to segregate multiple sound sources [1]. Although human psychophysical studies suggest ITDs can be detected in the fine structure of low-frequency sounds (<1.5 kHz) and in the modulating envelope of high-frequency complex sounds, ITD sensitivity is typically better for lower frequencies [2], [3]. Nonetheless, evidence suggests that normal-hearing listeners are sensitive to ITDs within sinusoidal amplitude-modulated (SAM) tonal carriers [4]. Furthermore, recent evidence from human psychophysical studies suggests that envelope ITD sensitivity can be enhanced using HWR envelopes [5], [6], [7], [8], [9]. Compared with SAM stimuli, HWR envelopes have longer gaps between modulating envelopes and a steeper rise time for each envelope.

This is of potential clinical interest to cochlear implant (CI) recipients, since this may provide a method to enhance binaural sensitivity. Until recently, CIs have been inserted in one ear only. Since recipients of unilateral CIs have particular difficultly localizing sounds and detecting speech in background noise, CIs in both ears have been trialled worldwide and, in some countries, have become the standard of care for children with severe to profound hearing loss. However, results suggest that ITD sensitivity is generally poor among CI recipients that may significantly limit potential benefits of bilateral CIs [10], [11], [12], [13], [14].

A recent study by Laback and colleagues [8] suggested that envelope shape is important for ITD sensitivity in deaf individuals with bilateral CIs. Specifically, they showed that envelope-ITD sensitivity may be improved for CI recipients by enhancing the signal envelope. Evidence from lesion studies suggests the importance of a functioning auditory cortex to binaural sensitivity [15]. In the current study, we assessed the effects of envelope shape using HWR stimuli in an animal model. Specifically, we found that envelope enhancement increased cortical sensitivity to ITDs in normal-hearing animals and bilateral profoundly-deaf animals with CIs in both ears.

## Materials and Methods

### Animal preparation

Twelve adult pigmented ferrets (*Mustela putorius*) were used in this study: 7 normal-hearing animals and 5 deaf animals with bilateral cochlear implants. This study was carried out in strict accordance with local ethics guidelines (approved by Oxford University Committee for Animal Care and Ethical Review), the UK Home Office Animals (Scientific Procedures) Act 1986 and under a personal Home Office license.

Otoscopy was performed before the experiment to make sure the external ear canals were free of wax and disease. Anesthesia was induced with intramuscular administration of medetomidine hydrochloride (Domitor; 0.08 mg/kg; Pfizer, Sandwich, UK). On induction, an intramuscular injection of atropine sulphate (0.06 mg/kg, C-Vet Veterinary Products, Leyland, UK) was given to minimize airway secretions. A 24-gauge cannula was inserted into a peripheral vein and anesthesia was maintained with a continuous infusion of a mixture of medetomidine hydrochloride (0.02 mg/kg/h) and ketamine (Ketaset; 5 mg/kg/h; Fort Dodge Animal Health, Southampton, UK) in 5% glucose/saline solution. The infusion was supplemented with 0.5 mg/kg/h dexamethasone (Dexadreson; Intervet UK Ltd, Milton Keynes, UK) and 0.06 mg/kg/h atropine sulfate to minimize cerebral edema and bronchial secretions, respectively. The animal was intubated with an endotracheal tube through which the animal was ventilated. Core body temperature, end-tidal CO_2_, and electrocardiogram were monitored throughout the experiment. A steel head bar was attached to the skull with stainless-steel screws and dental cement to fix the animal’s head. A craniotomy was performed to expose the left auditory cortex, the dura was removed and cortical motion was reduced with a thin layer of 1.5% agar in saline.

### Acoustic stimulation in normal-hearing animals

For normal-hearing animals (n = 7), acoustic stimuli were generated using Tucker-Davis Technologies (TDT, Alachua, FL) System III hardware and TDT Brainware and Real Time Processor Visual Design Studio (RPvdsEx) software (50-kHz sampling rate). Subsequently, they were presented binaurally over earphones (Panasonic RP-HV297, Bracknell, UK) coupled to otoscope specula that were inserted into both ear canals. The transfer function of the earphones was canceled from the stimulus using an inverse filter to ensure the frequency response of the drivers was flat from 0.5 to 25 kHz (±2-dB SPL). Closed-field calibration was performed using a 1/8 inch condenser microphone (Type 4138, Brüel & Kjær UK, Stevenage, UK).

#### Characterization stimuli

Broadband noise (70-dB SPL; 0.05- to 30-kHz spectral range; 100-ms duration with a 5-ms cos^2^ rise/fall time) presented to both ears was used as a search stimulus in order to establish whether a unit was acoustically responsive. Subsequently, the frequency tuning of each acoustically-responsive unit was characterized with tones (100-ms duration with 10-ms rise-fall times) presented to both ears over a range of frequencies (0.4 to 22 kHz in 12 logarithmic steps) and levels (60- to 80-dB SPL in 10-dB steps). The best frequency (BF) of a unit was defined as the tone frequency that elicited the largest number of responses within a 500-ms recording window after the onset of the stimulus. Envelope ITD sensitivity was assessed in all driven units using stimuli that are described in detail below.

#### Bilateral acoustic stimulation

**Figure 1:**
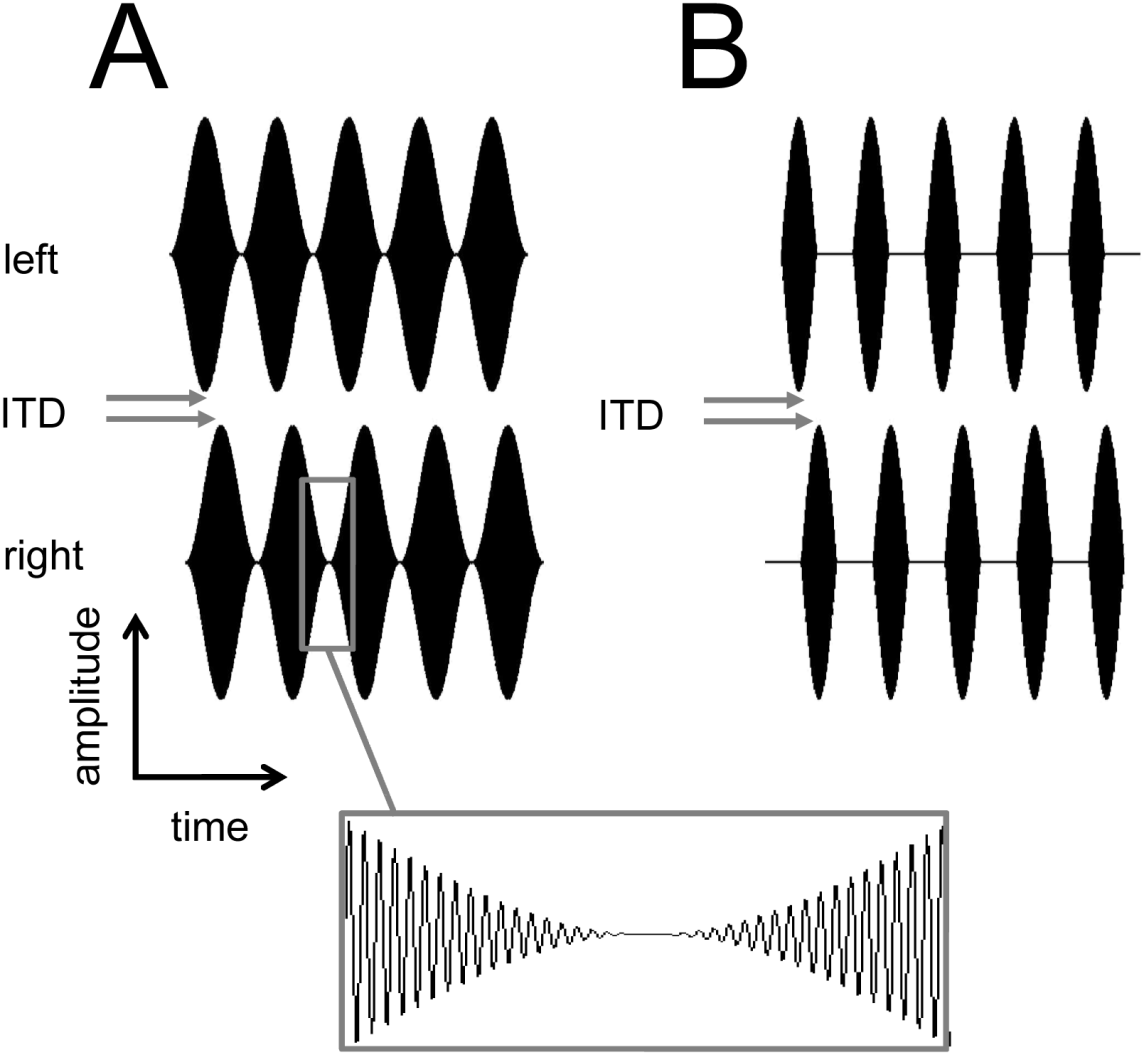
Acoustic stimuli presented to normal-hearing animals. Time waveforms illustrating the acoustic stimuli that were presented, including SAM (A) and HWR tones (B). An interaural time delay (ITD) was created by delaying the envelope in one channel and advancing it in the other by an equal amount. The box shows a segment of the stimulus envelope and fine structure in more detail.

In the guinea-pig, it has previously been shown that neural sensitivity to envelope ITDs is limited to below approximately 600 Hz modulation frequency [16], [17] using both SAM and HWR stimuli. In the same species, it has been shown that neurons can respond most strongly to ITDs that occur outside the normal physiological range of ITDs [18], [19]. In the current study, cortical sensitivity to envelope ITDs was measured using tones presented at the BF of the unit, amplitude modulated with SAM and HWR envelopes over a range of modulation frequencies (150–600 Hz in 150 Hz steps; 100% modulated; 70 dB SPL rms level; 100-ms duration; Figure 1). The average BF across the population of recorded units was 8 kHz (SD 4.9). The modulation frequency range was chosen to include values at which sensitivity to ITDs has been previously observed at high frequencies [4], [20]. The ITDs used in the current study (±1 ms in 0.1-ms steps) were chosen to extend beyond the range of values that a ferret would normally encounter within its free-field acoustic environment (approximately ±0.2–0.3 ms), based on previous studies in our laboratory [21]. Specifically, the envelope of the stimulus contained onset-, ongoing- and offset-ITDs, whilst the fine structure of the tonal carrier remained in phase (at zero delay) between the ears. A positive ITD was generated by delaying the stimulus in the ipsilateral ear to the recording site (left) and advancing the stimulus in the contralateral ear. Negative ITDs were generated by delaying the stimulus on the contralateral (right) side and advancing the ipsilateral stimulus. All stimuli were gated with 5-ms cosine-squared rise-fall times in each ear.

For each modulation condition (SAM and HWR), there were 84 stimulus parameter combinations presented 15 times each in a pseudo-random order. Stimuli were presented at a rate of one every second. The basic protocol required approximately 40 minutes. For most units the protocol could be completed in its entirety without losing any responsiveness.

### Intra-cochlear electrical stimulation in deaf animals

#### Deafening and cochlear implantation

Our methods for deafening and cochlear implantation have been described in more detail elsewhere [22]. Briefly, adult ferrets (n = 5) were deafened with intra-scalar aminoglycoside administration and implanted with a custom-made intra-cochlear electrode array in each ear. Under general anesthesia, a hole was drilled into the tympanic bulla through a post-aural incision to expose the round window membrane. After the round window was opened with a fine needle, the scala tympani was slowly irrigated (over 5 minutes) with approximately 0.5 ml of neomycin sulphate (10 mg/ml in normal saline; Pfizer, Sandwich, UK). This deafening procedure was repeated in the contralateral ear and profound bilateral hearing loss was confirmed in all animals through the absence of an auditory brainstem response to acoustic clicks presented at ≥95 dB SPL.

After deafening, a custom-made intra-cochlear electrode array was inserted through the round window of each ear to an approximate depth of 7.5 mm into the scala tympani. The electrode array, fabricated using injection molding techniques, consisted of three platinum ring electrodes at its tip (∼0.43 mm in diameter with an inter-electrode separation of ∼0.5 mm). All three electrodes were implanted inside the cochlea, followed by two markers just proximal to the last electrode spaced by 0.2 mm. Using these markers to judge the depth of implantation ensured place-matched intracochlear electrodes for each bilateral CI procedure.

#### Electrically-evoked auditory brainstem responses (EABRs)

**Figure 2:**
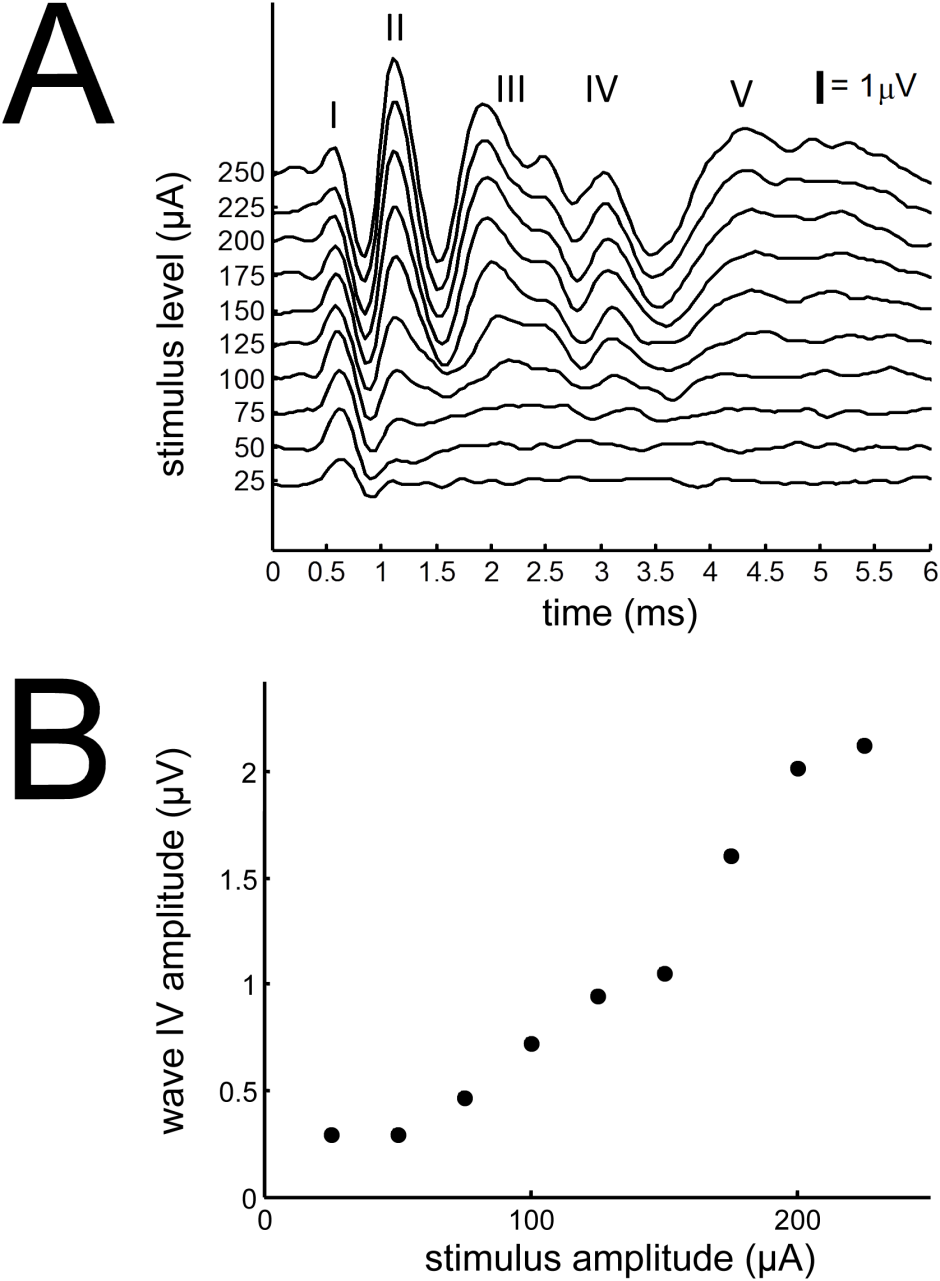
Electrically-evoked auditory brainstem responses from a deaf animal with cochlear implants. (A) Representative electrically-evoked auditory brainstem responses (EABR) plotted for each stimulus level. The black bar represents 1 µV. (B) Amplitude of wave IV of the EABR plotted against stimulus level.

Hearing thresholds can vary depending upon the position of the intra-cochlear electrode array [23]. Therefore, EABRs were recorded to estimate neural thresholds for intra-cochlear electrical stimulation in each ear separately (Figure 2). Throughout these recordings the animal’s temperature was maintained at 37.5±1°C and depth of anesthesia was assessed with withdrawal to paw pinch. EABR responses were recorded differentially using subcutaneous needles. Needles positioned at the vertex, inion and thorax were used as positive, negative and ground electrodes, respectively. Responses were amplified and digitally filtered (once forward and once reversed) with a 31^st^-order finite impulse response bandpass filter (0.3–3 kHz pass band). Electrical stimuli were generated using Tucker-Davis Technologies (TDT, Alachua, FL) System III hardware and Real Time Processor Visual Design Studio (RPvdsEx) software (25-kHz sampling rate). Specifically, biphasic current pulses (40 µs/phase; 10/s repetition rate) with alternating polarity were delivered to the intra-cochlear electrode array in a wide-bipolar configuration (∼1.5 mm between the apical, active electrode and the basal, return electrode). Pulses were presented over a range of levels (25 µA to 750 µA, in 25 µA steps) in a pseudorandom order and repeated five hundred times. The stimulus artifact alternates in polarity with the stimulus, whereas the neural response does not. Thus, the artifact was largely averaged out from the summed neural response [24]. The EABR threshold was defined as the minimum stimulus intensity producing a response amplitude of at least 0.4 µV for wave IV (latency window of 3–3.5 ms following the stimulus onset).

#### Bilateral cochlear implant stimulation

**Figure 3:**
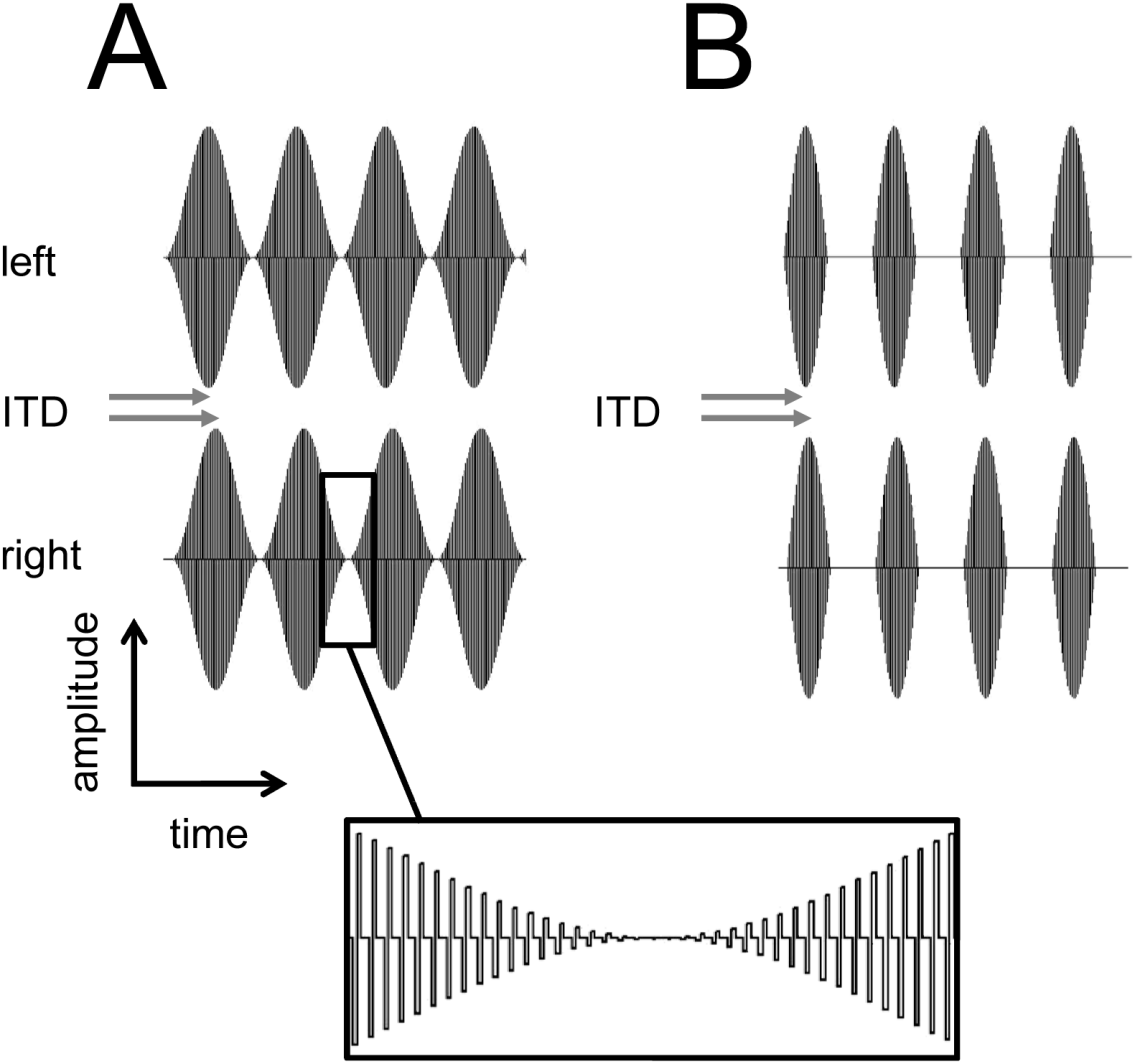
Electrical stimuli presented to deaf animals with cochlear implants. Time waveforms of the electrical stimuli that were presented, including SAM (A) and HWR biphasic-pulse trains (B). The box shows a segment of the stimulus envelope and fine structure in more detail.

In deaf cats, envelope ITD sensitivity of inferior colliculus (IC) neurons has been demonstrated using SAM pulse trains with a carrier frequency of 5000 pulses per second [25]. That study also showed that fine structure ITD sensitivity was rarely observed at this carrier rate. In the current experiment, we measured cortical ITD sensitivity in response to biphasic pulse trains (cathodic-anodic, 40 µs/phase, 6000 pulses per second, 100-ms duration) with SAM or HWR envelopes (Figure 3). These pulse trains were delivered to the intracochlear electrode arrays in each ear in wide bipolar configuration at 3 dB above the EABR threshold in each ear. As for acoustic stimuli, envelope ITDs (±1 ms in 0.1-ms steps) were generated over a range of modulation frequencies (150–600 Hz in 150 Hz steps; 100% modulated). Again, whilst the envelope of the stimulus contained onset-, ongoing- and offset-ITDs, each pulse within the train remained in phase (at zero delay) between the ears. There were 84 stimulus parameter combinations for each modulation condition (SAM and HWR). Stimuli were presented every second and repeated 15 times in a pseudo-random order.

### Electrophysiological recordings

Extracellular single- and small multi-unit cluster activity was recorded within a sound-attenuated chamber (Industrial Acoustics Company, Winchester, UK) using single-shank silicon probes with 16 recording sites (NeuroNexus Technologies, Ann Arbor, MI; inter-site separation 100 µm). All recordings were band-pass filtered (0.5 to 5 kHz), amplified and digitized at 25 kHz. Stimulus generation and data acquisition were synchronized using Brainware software (Tucker-Davis Technologies). Data were acquired in 1 second sweeps triggered by the onset of the stimulus. Any recorded event with a magnitude of 2.5 times the mean of the recorded amplitude of the raw waveforms was considered to be a potential spike. The latencies and shapes of all potential spikes were stored for offline analysis. For each animal, we recorded activity from approximately 10–40 evenly-spaced recording sites within the primary auditory cortex. Auditory cortex was targeted using sulcal landmarks according to the criteria of Bizley and colleagues [26].

**Figure 4:**
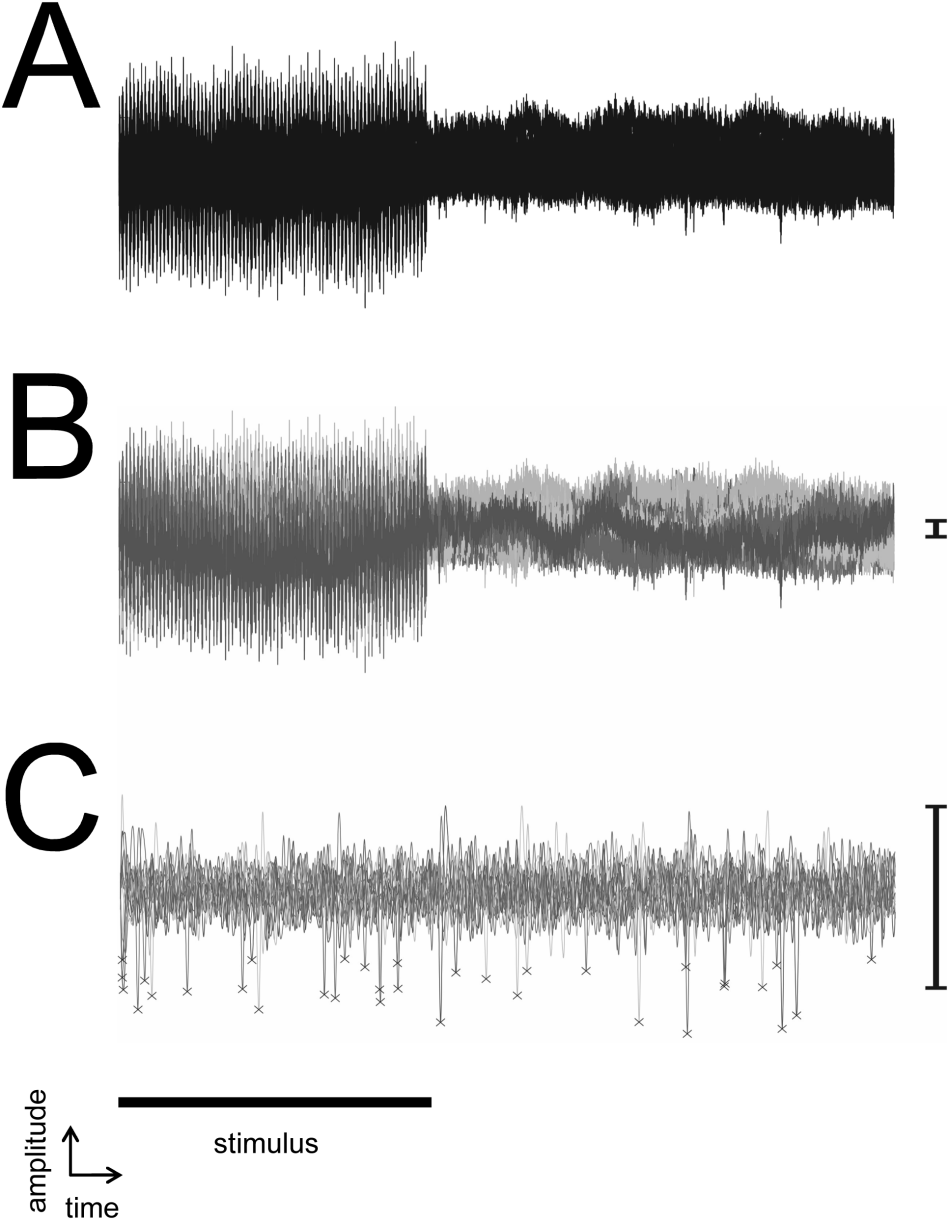
Stimulus artifact. (A) Example of a cortical recording in response to bilateral intra-cochlear electrical stimulation. The stimulus artifact is coincident in time with the stimulus. (B) Responses containing artifact were sub-classified using artifact rejection methods (plotted using different shades of line). (C) Example of recording following artifact rejection. Crosses mark probable neural responses. Low spike counts suggest that neural responses may have been removed with the artifact rejection method. The bar to the right of B & C represents the same amplitude and the stimulus artifact is significantly larger than the neural response.

In animals with CIs, cortical electrophysiological recordings were complicated by artifacts derived from bilateral intra-cochlear electrical stimulation (Figure 4). Pilot studies revealed that artifacts could be reduced, but not eradicated, from these recordings using artifact rejection techniques [27]. However, these techniques may also underestimate cortical-evoked activity. On average, the spike rate in response to a 100-ms pulse train was about ten times lower than would be predicted based on results from single-pulse stimulation. This suggests that our rejection methods removed a significant proportion of neural responses along with the artifact. Furthermore, the electrical artifact varied with ITD, since it was derived from a combination of stimulating both ears. In theory, the proportion of neural responses removed through artifact rejection could also vary with ITD. This could create a further bias in the analysis of binaural electrically-evoked responses. Subsequently, an alternative method of artifact avoidance was used. In response to stimuli of ≥100 ms, distinct cortical responses occur after the onset and the offset of the stimulus. Furthermore, binaural sensitivity is frequently observed in both onset and offset responses [28]. In all subsequent experiments we delayed the recording until the electrical stimulus was undetectable. Thus only offset responses were recorded in animals with CIs. Since acoustic stimulation is not associated with stimulus artifact, both onset and offset responses were recorded in normal-hearing animals.

### Data Analysis

#### Spike sorting

Spike sorting algorithms were based on those used in previous studies within our laboratory [28]. Spikes were sorted off-line using a ‘k-means clustering’ algorithm incorporated into Brainware. Specifically, clusters were chosen on the basis of spike shapes. Subsequently, a test of the refractory period in the auto-correlation histogram was used to assess whether the chosen cluster was more likely to be a single- or multi-unit. Any cluster containing <1% of spikes with an inter-spike interval of <1.5 ms was classed as a single-unit, whist the remaining clusters were classed as small multi-units. Spike times were then exported into Matlab 7.0.1 (The MathWorks, Natick, MA) for further analysis.

#### Response period

**Figure 5:**
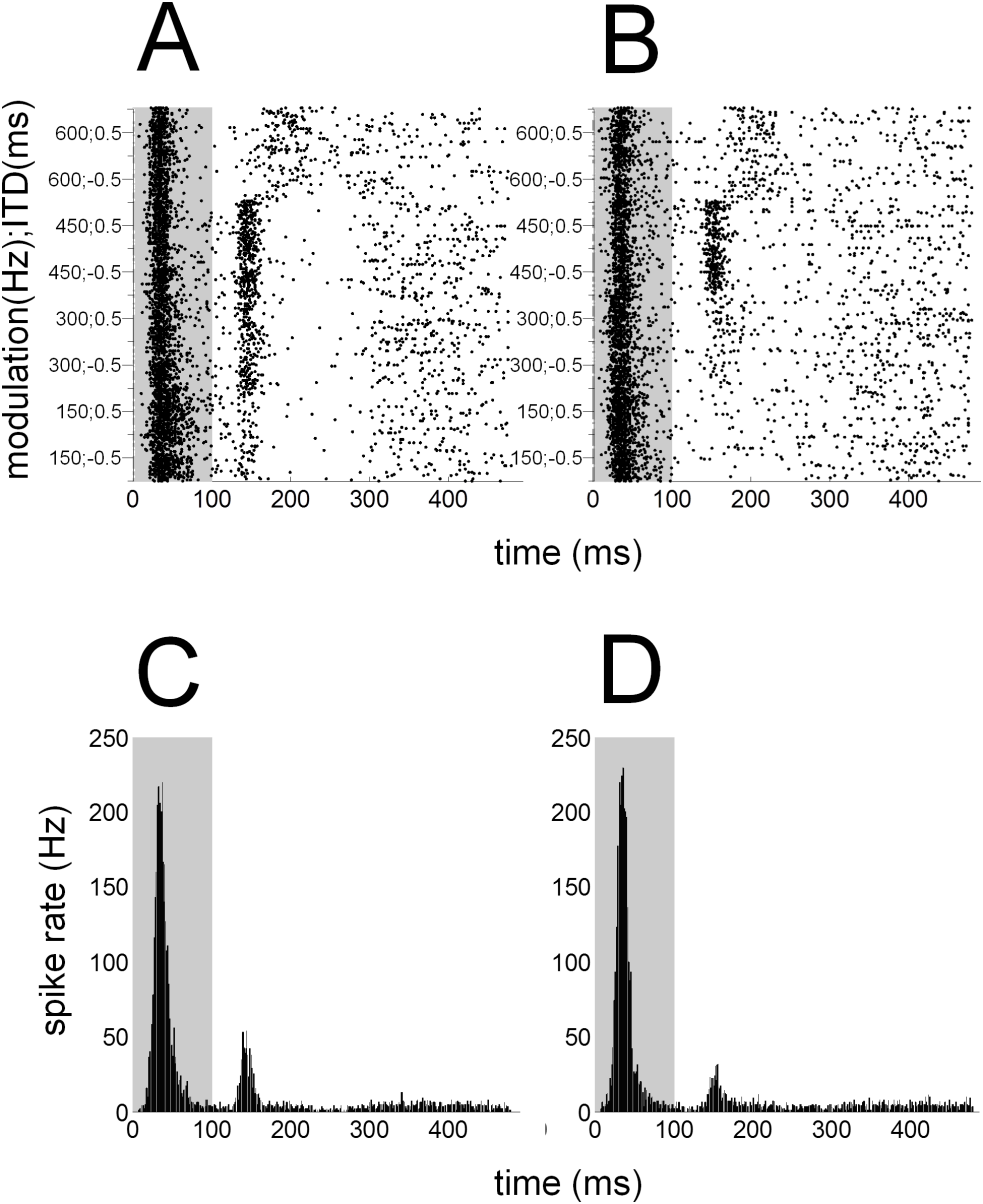
Cortical responses from a normal-hearing animal. Example of dot-raster plots (A & B) and post-stimulus time histograms (C & D) for an individual unit with a significant response to both SAM (A & C) and HWR tones (B & D). The stimulus duration is represented by the light grey bar. Dot-raster plots (A & B) show spike timing as black dots, with time and stimulus plotted on the x- and y-axes, respectively. Along the y-axis the different stimuli are arranged from higher- to lower-frequency of modulation and from positive to negative ITDs.

**Figure 6:**
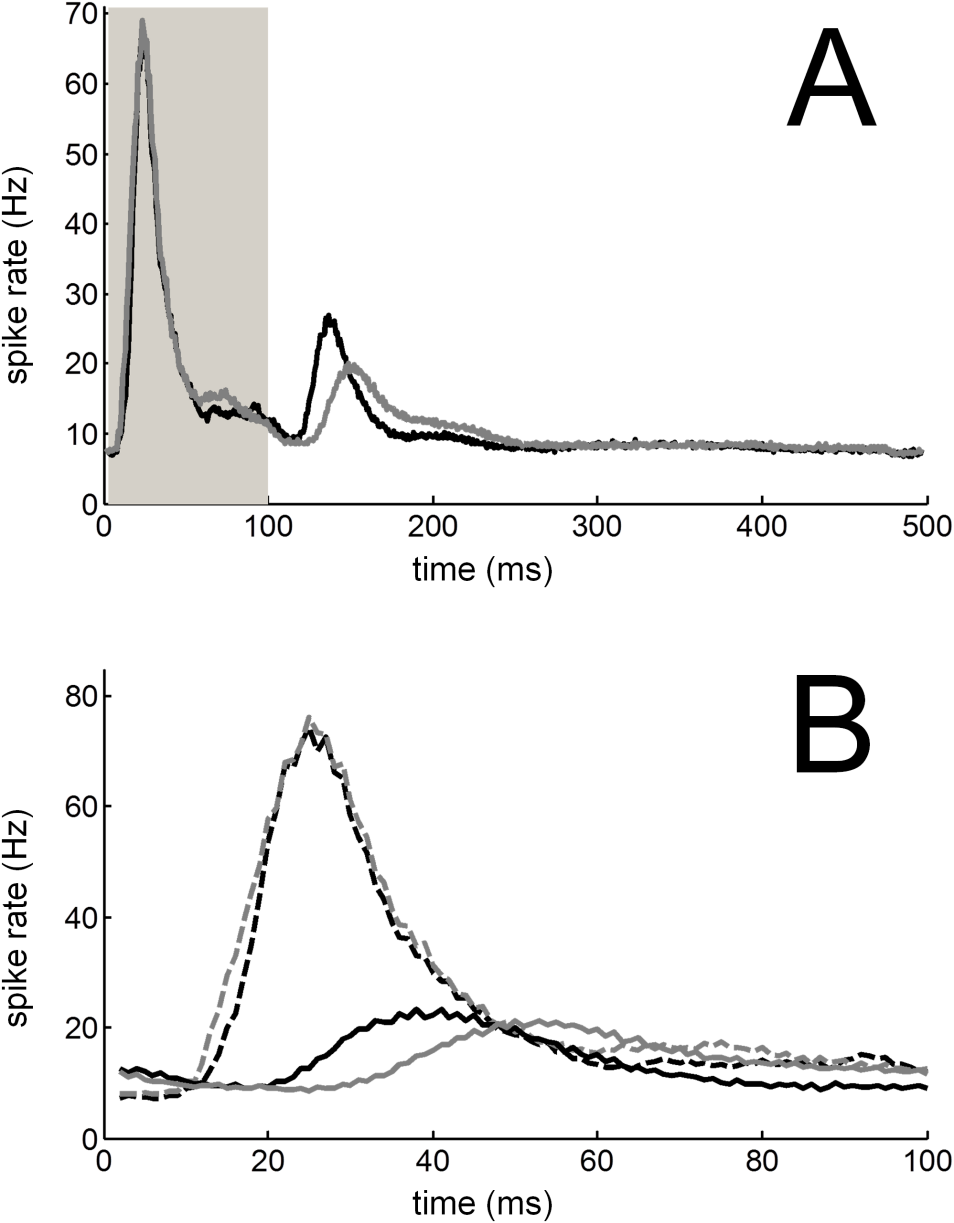
Average post-stimulus time histogram from all normal-hearing animals. (A) Average post-stimulus time histogram across the population of units in response to SAM (black line) and HWR tones (grey line). The stimulus duration is represented by the light grey bar. (B) Mean spike rate in response to SAM (black lines) and HWR tones (grey lines) across all units, for the onset- (dashed lines) and offset-response windows (solid lines), are superimposed by aligning the stimulus onset and offset at time = 0 on the abscissa.

Initially for each recording, a dot raster and peri-stimulus time histogram (PSTH) with a 1-ms bin width was constructed by counting the number of spikes in response to each trial (Figure 5). Similar to previous studies in our laboratory [28], excitatory responses in A1 were classified based on the discharge patterns. For normal-hearing and deaf animals, spikes were averaged using a 70-ms duration window positioned between 5 and 75 ms after the offset of the stimulus. Since onset responses could be recorded in hearing animals, we also averaged spikes between 5 to 75 ms after the onset of the stimulus in those animals. These windows were chosen following initial analysis of the peak latency of on and off responses across our population of neurons (Figure 6). The background or spontaneous spike rate for each recording was calculated from the PSTH by averaging the spikes occurring between 5 and 75 ms before the stimulus onset. Using a Poisson cumulative distribution function an excitatory response in the onset and/or offset period was defined as ‘driven’ if the spike rate in the response period was greater than that of the spike rate in a spontaneous window with a probability of ≥0.99.

#### Binaural sensitivity

In units with a significant onset and/or offset response to both modulation conditions (SAM and HWR), ITD sensitivity was assessed using methods previously described in detail in an earlier publication from our laboratory [28]. Briefly, binaural sensitivity was initially assessed by determining whether Poisson regression models of up to 4^th^ order fitted the data significantly better than a null model that assumed no effect of the stimulus. A likelihood ratio test was performed to determine whether the Poisson regression model fitted the data significantly better than the null model at P<0.05. A generalized linear model (Statistics Toolbox, Matlab, Natick, MA) was used to express the observed spike count (y) as a function of stimulus parameter (x) in the form
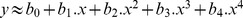
where *b_0_, …, b_4_* are free parameters. These polynomials can approximate the non-linearity associated with physiological recordings such as saturation, non-monotonicity and thresholding. Furthermore, they make few assumptions about the shape of the ITD response function to capture the diversity of observed binaural responses in cortical neurons [28]. Separately, a null model was fitted to the data using the formula



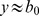



This null model assumes that the spike count (y) randomly varies around a spontaneous rate of *b_0_*, independent of the stimulus. Subsequently, a likelihood ratio test was used to determine whether the Poisson regression model fitted the data significantly better than the null model at P<0.05, by computing the deviance for the two models. The deviance is equivalent to minus twice the difference between the log-likelihoods of the fits of the two models to the data and is approximately a *χ^2^*-distribution with degrees of freedom equal to the order of the regression model.

In the same units, an index of ITD sensitivity (d’) was also calculated across all recording sweeps using the formula:
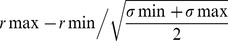



where i) *r* max and *r* min represent the maximum and minimum firing rate averaged across all repeat presentations of the same stimulus, respectively, and ii) σ min and σ max represent the corresponding standard deviations [29]. In response to both modulation conditions (SAM and HWR) the index of ITD sensitivity was compared using analysis of variance.

## Results

These results are based on 592 single and small multi-unit clusters recorded from the left auditory cortex of 12 adult ferrets. Specifically, 448 units were recorded from 7 normal-hearing ferrets and 144 units were recorded from 5 profoundly-deaf ferrets with bilateral CIs.

### Timescale of cortical responses in normal-hearing ferrets

Initially, for each recording a dot raster and PSTH with binwidth of 1 ms was constructed by counting the number of spikes in response to each trial (Figure 5). During subsequent analysis, the average PSTH for the population of units was plotted for responses to SAM and HWR stimuli (Figure 6). In response to both stimulus conditions, the mean PSTH revealed excitatory responses that occurred predominantly after the onset and the offset of the stimulus. A threshold was used to classify recordings that had a maximal firing rate significantly higher than the spontaneous firing rate of the unit within the onset- or offset-response window. This analysis revealed that 416 and 336 units had significant on and off responses to acoustic stimulation, respectively.

To compare the time-course of on and off responses (Figure 6A), mean responses to SAM and HWR stimuli were superimposed by aligning the stimulus onset and offset at time 0 (Figure 6B). Compared with off responses, on responses were characterized by a shorter rise time (‘peak latency’). On average, the peak latency for on responses was 25 ms relative to the sound onset, regardless of the stimulus type (SD 17.7 and 14.1 for the SAM and HWR conditions respectively; (Figure 6B). On average, the offset peak latency to HWR stimuli was longer than the equivalent response to SAM stimuli. Specifically, the average peak latency of the off response to SAM and HWR stimuli was 38 ms (SD 57.3) and 53 ms (SD 60.1) relative to the sound offset respectively.

Consistent with previous results from our laboratory [28], the ‘peak spike rate’ of the off response to both stimulus conditions was less than half that of the on response (Figure 6B). Also the onset response decayed to half of the peak rate (‘half decay time’) at about the same time, regardless of the stimulus condition (37 ms, SD 18.2 and 39 ms, SD 15.2 after the onset of SAM and HWR stimuli respectively). Compared with onset responses, offset responses took longer to decay to half of the peak value. Furthermore, the offset half-decay time was longer in response to HWR (122 ms after the sound offset, SD 64.8) compared with SAM stimuli (69 ms after the sound offset, SD 60.5; Figure 6B).

### Binaural sensitivity in normal-hearing ferrets

**Figure 7:**
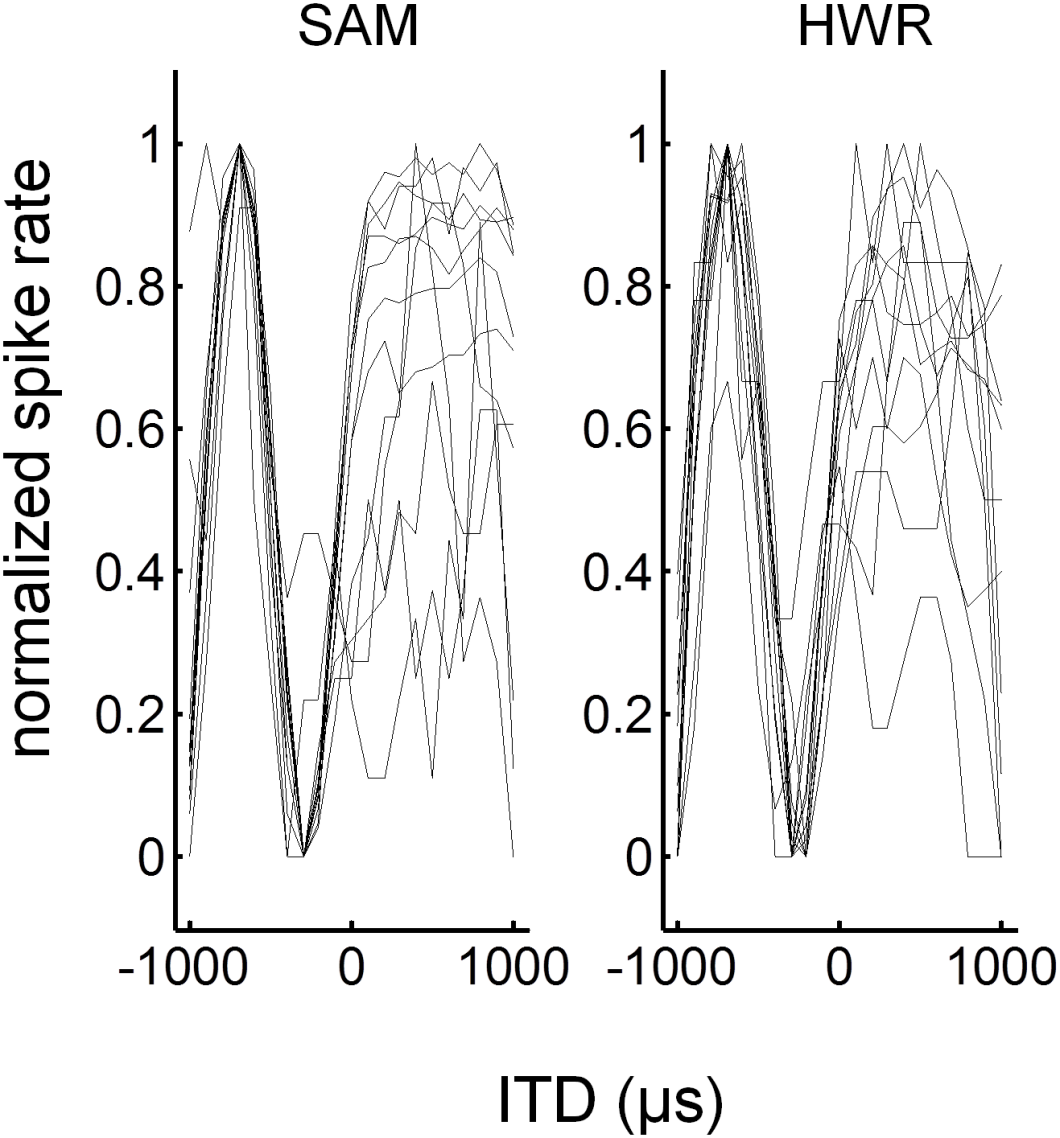
ITD functions in normal-hearing animals. Examples of normalized ITD functions in response to SAM and HWR tones. Each ITD response function was derived from evenly spaced recording locations along the same electrode penetration, positioned orthogonal to the cortical surface.

**Figure 8:**
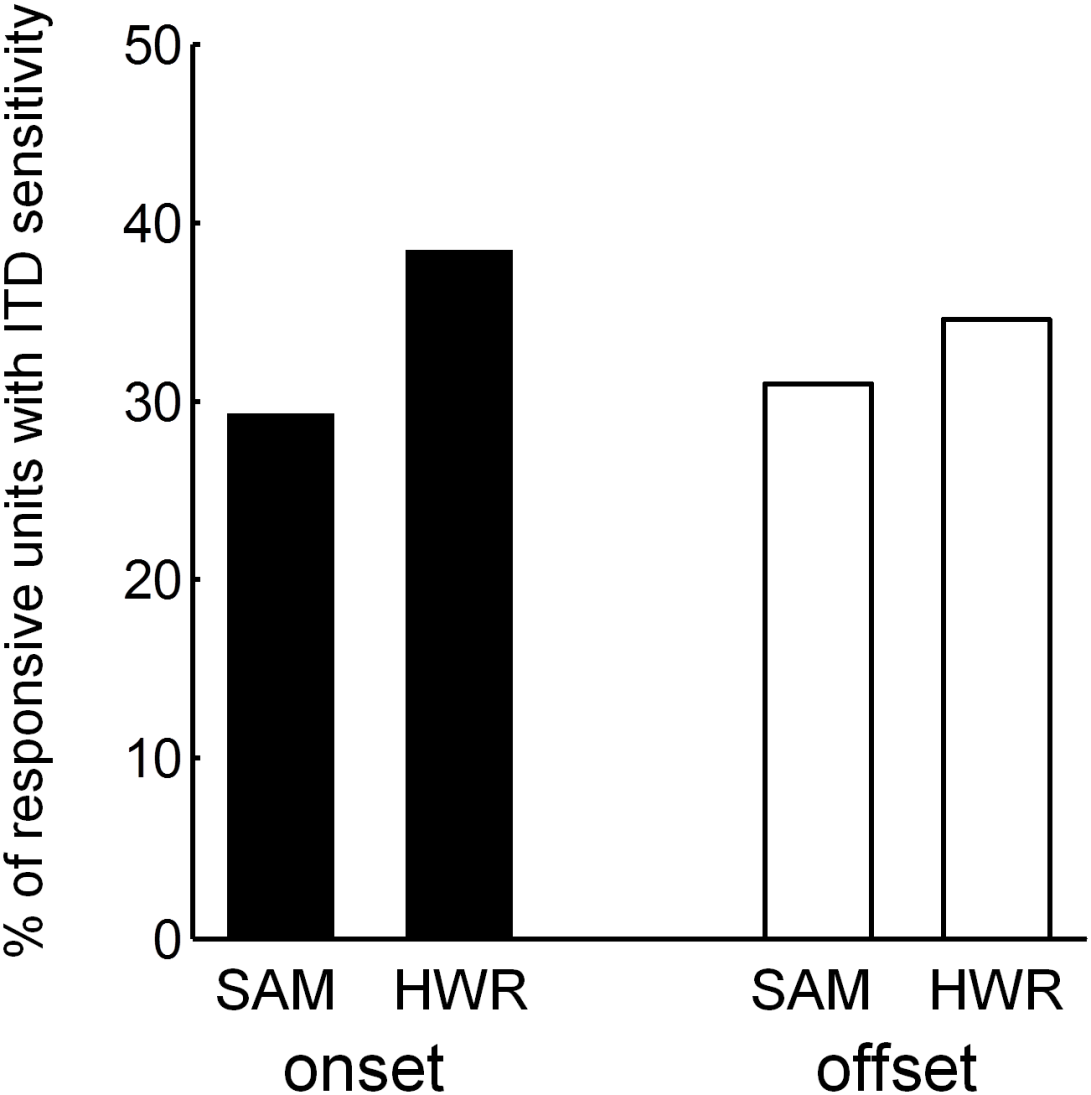
ITD sensitive units in normal-hearing animals. Percentage of responsive units with ITD sensitivity within the onset and offset response to SAM and HWR tones.

ITD response functions to SAM and HWR stimuli (Figure 7) were generated from mean spike rates averaged across each recording window. A likelihood ratio test was used to assess ITD sensitivity by determining whether a Poisson-regression model up to 4^th^ order fitted the data significantly better than the null model at P<0.05. Of the units that had an on response (416 units) more units were ITD sensitive, as defined by the significance level test, in response to HWR (160 units = 38%), compared with SAM (122 units = 29%) stimuli (Figure 8). Likewise, of the units that had a significant off response (336 units) more units were ITD sensitive in response to HWR (116 units = 35%), compared with SAM (101 units = 30%) stimuli.

**Figure 9:**
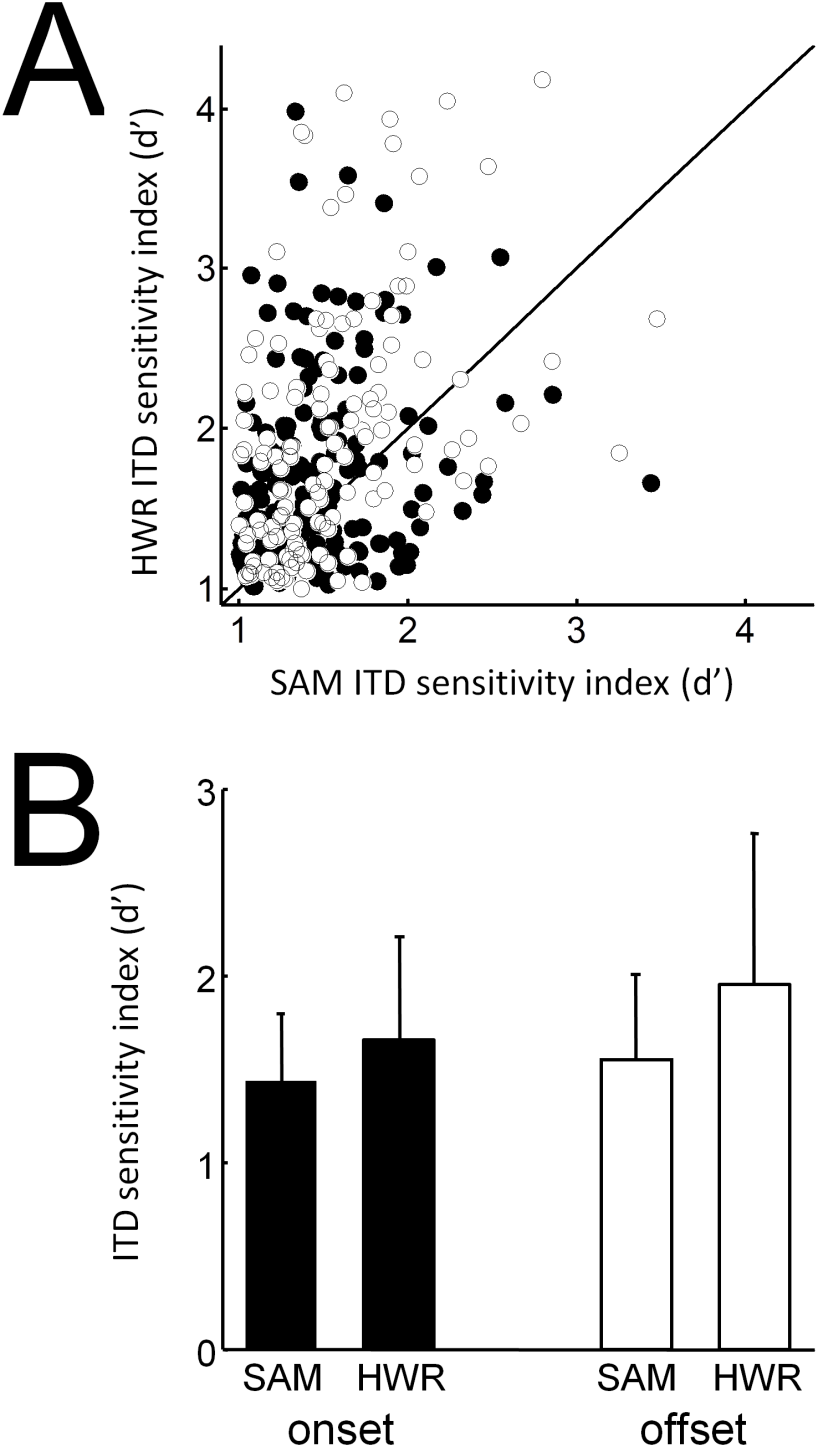
ITD sensitivity index in normal-hearing animals. (A) ITD sensitivity index for individual units plotted for onset (closed circles) and offset (open circles) responses to SAM and HWR tones. (B) Mean ITD sensitivity index (± SD) plotted against stimulus condition for onset and offset responses.

Figure 9A plots the ITD sensitivity index for on and off responses. The mean ITD sensitivity index was higher i) in response to HWR, compared with SAM stimuli and ii) for off responses, compared with on responses (Figure 9B). Specifically the mean ITD sensitivity index was lowest for on responses to SAM stimuli and highest for off responses to HWR stimuli. A two-way analysis of variance revealed i) a significant main effect for stimulus condition (SAM vs. HWR; *F*_1, 11_ = 37.1, P<0.001) and response period (onset vs. offset; *F*_1, 8_ = 25.4, P<0.001) and ii) a significant interaction between stimulus condition and response period (*F*_1, 1_ = 5.0, P = 0.02).

**Figure 10:**
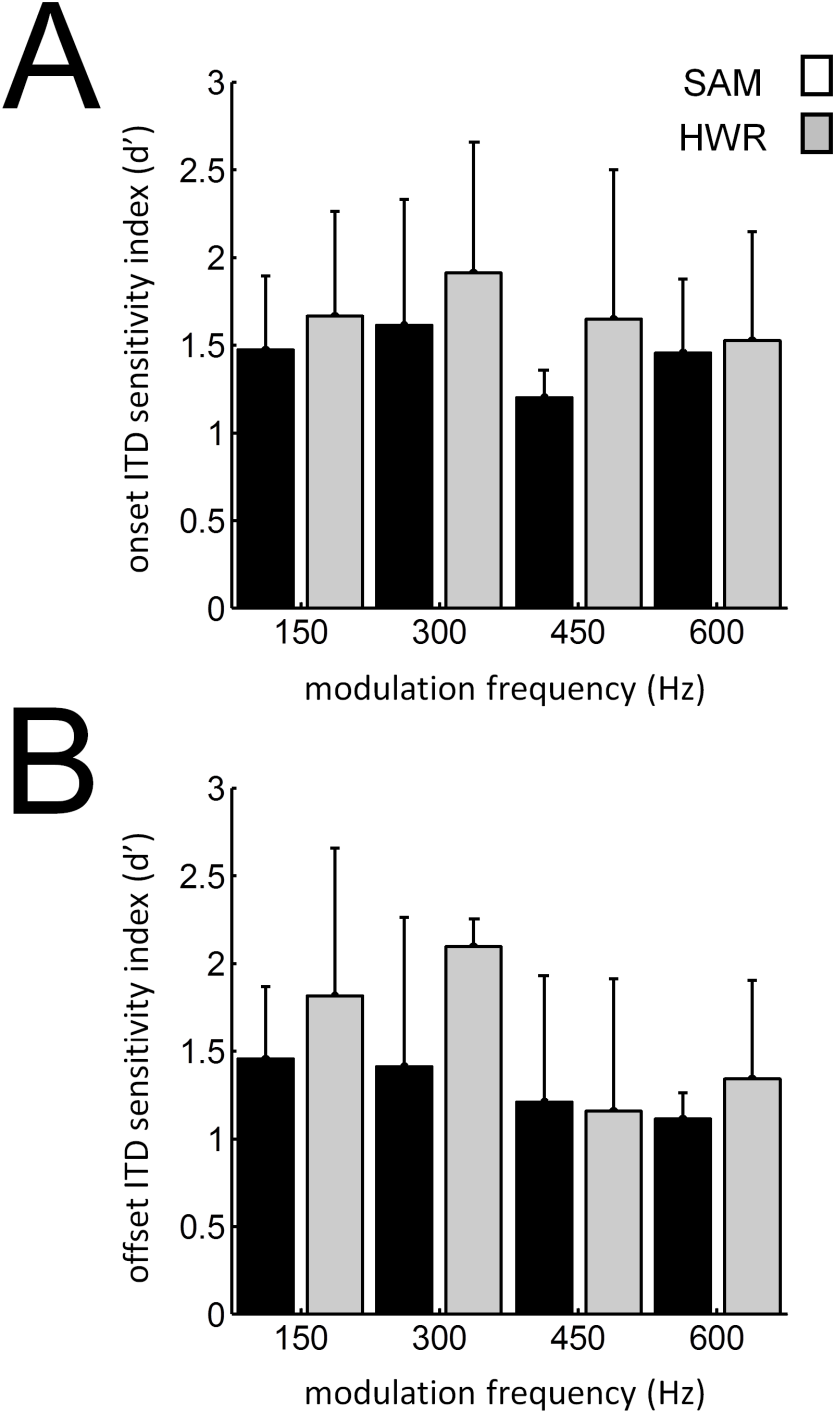
Average ITD sensitivity index for each modulation frequency in normal-hearing animals. Mean ITD sensitivity index (± SD) plotted against modulation frequency for (A) onset and (B) offset responses to SAM (black bars) and HWR tones (grey bars).

Consistent with previous data from our laboratory [28] we found that binaural sensitivity varied little with recording depth. Again, in agreement with this previous study we found no evidence that binaural sensitivity changed systematically across the surface of ferret auditory cortex. Furthermore, the mean ITD sensitivity index for on and off responses did not vary significantly between individual animals, nor did it vary significantly over the range of modulation frequencies tested (Figure 10).

### Timescale of cortical responses in deaf ferrets with bilateral CIs

**Figure 11:**
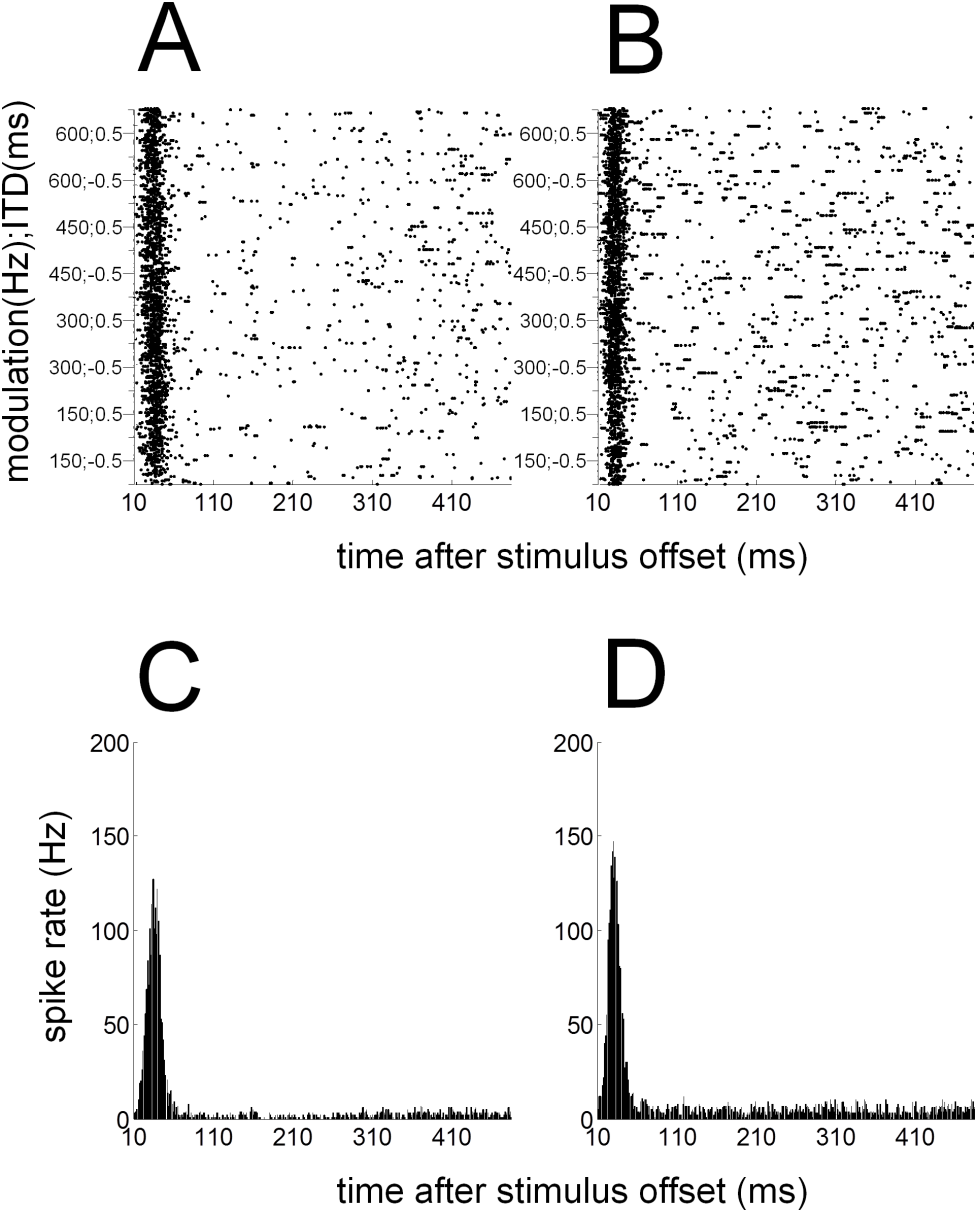
Cortical responses from a deaf animal with cochlear implants. Example of dot-raster plots (A & B) and post-stimulus time histograms (C & D) for an individual unit with a significant response to both SAM (A & C) and HWR pulse trains (B & D). Note the x-axis plots time after the stimulus offset. Dot-raster plots (A & B) show spike timing as black dots, with time and stimulus plotted on the x- and y-axes, respectively. Along the y-axis the different stimuli are arranged from higher- to lower-frequency of modulation and from positive to negative ITDs (as in Figure 5).

**Figure 12:**
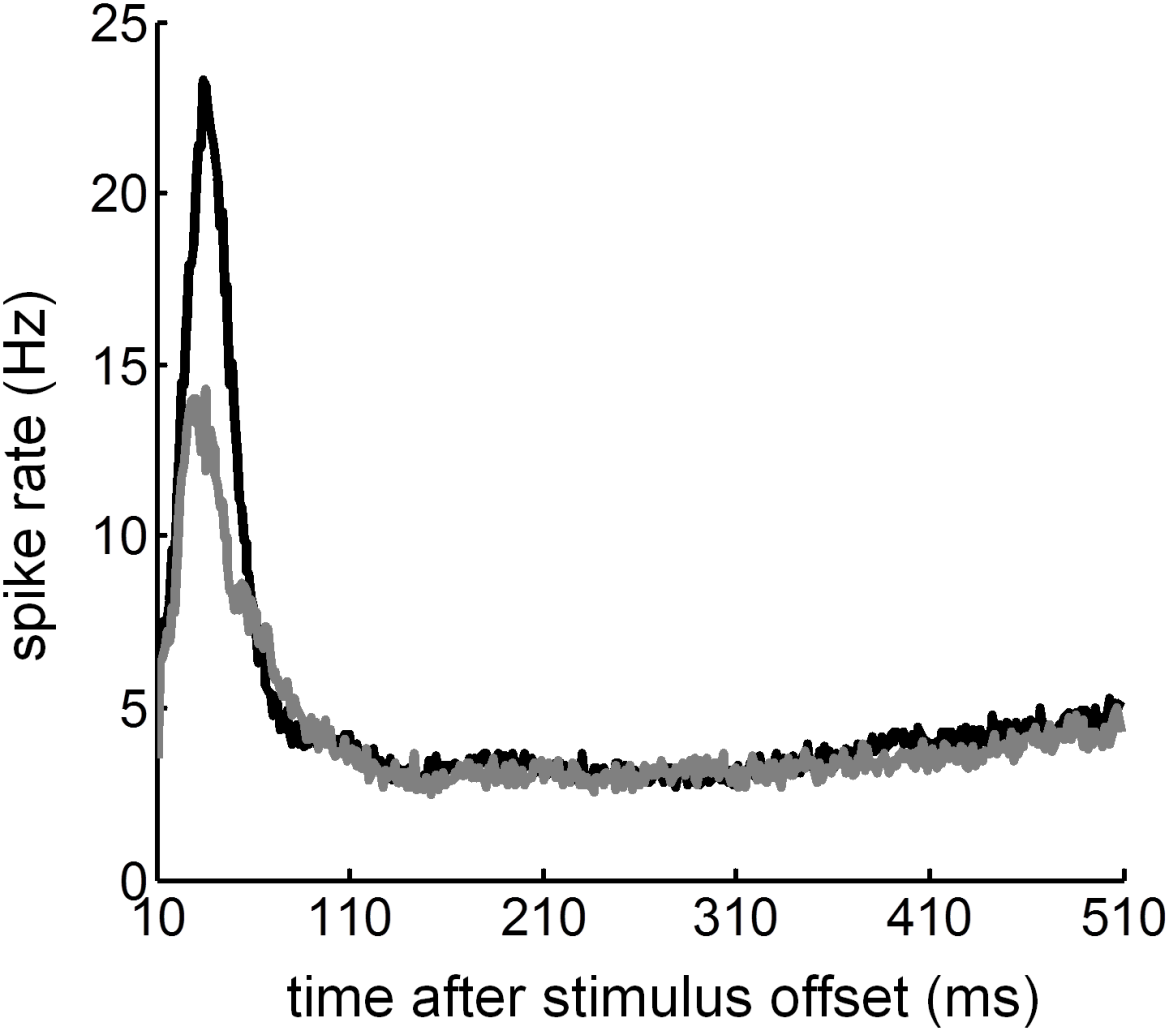
Average post-stimulus time histogram from deaf animals with cochlear implants. Average post-stimulus time histogram across the population of units in response to SAM (black line) and HWR pulse trains (grey line). Note the x-axis plots time after the stimulus offset.

Initially, a dot raster and PSTH were constructed for each recording (Figure 11). Subsequently, the average PSTH across the population of units was plotted for responses to SAM and HWR pulse trains (Figure 12). In response to both stimulus conditions the average PSTH revealed excitatory responses that occurred after the offset of the stimulus. Due to stimulus artifact the on response was not recorded. A threshold was used to classify recordings that had a significant peak in the PSTH above the spontaneous firing rate of the unit within the offset-response window. This analysis revealed that 112 units had significant off responses to intra-cochlear electrical stimulation. The average peak latency of the off response to SAM and HWR pulse trains was 36 ms (SD 18.2) and 29 ms (SD 19.9) relative to the sound offset, respectively. The offset half decay time was longer in response to HWR (168 ms after the sound offset, SD 18.8), compared with SAM pulse trains (154 ms after the sound offset, SD 20.2; Figure 12).

### Binaural sensitivity in deaf ferrets with bilateral CIs

**Figure 13:**
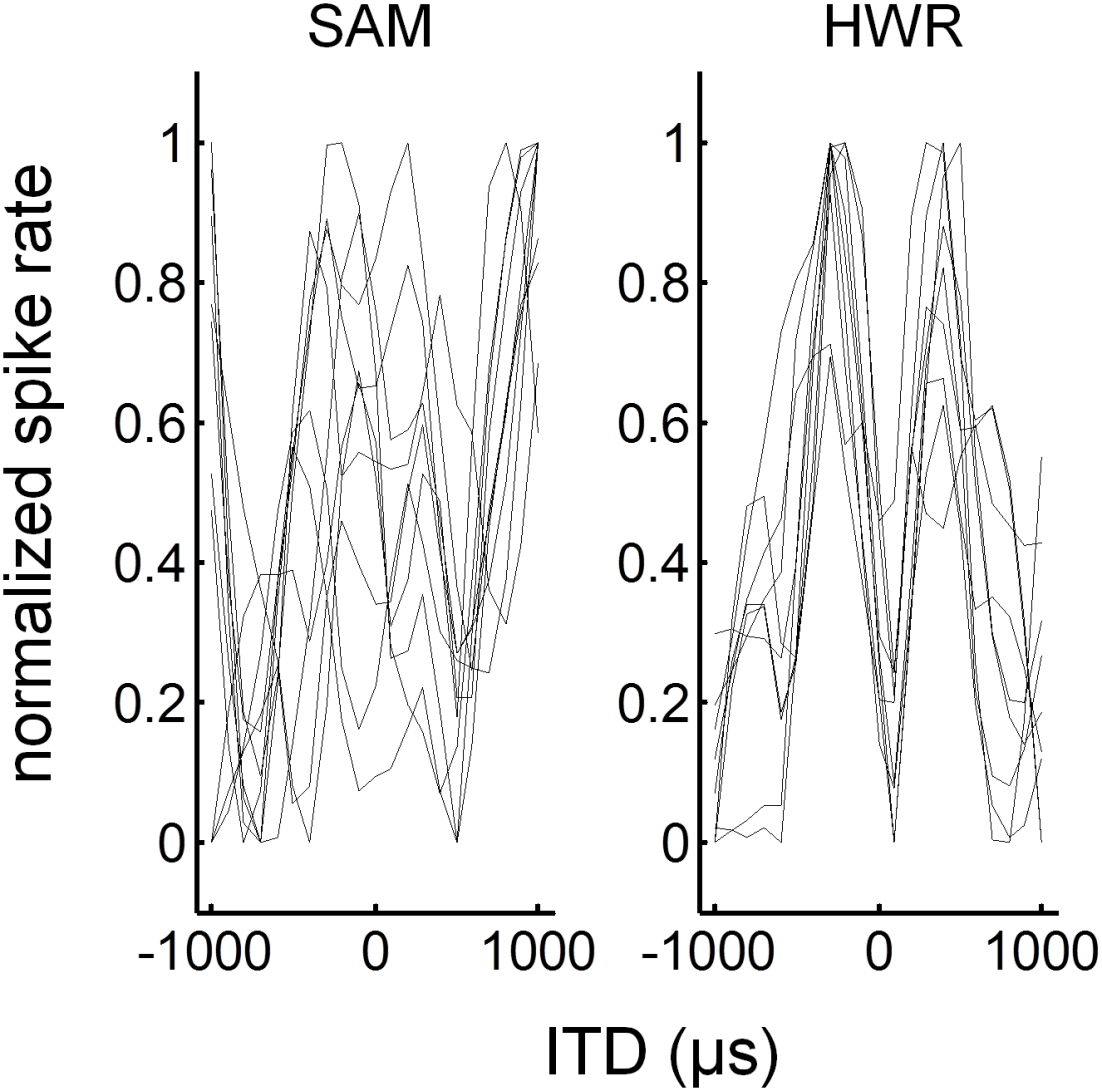
ITD functions in deaf animals with cochlear implants. Examples of normalized ITD functions in response to SAM and HWR pulse trains. Each ITD response function was derived from evenly spaced recording locations along the same electrode penetration, positioned orthogonal to the cortical surface (as in Figure 7).

ITD response functions to SAM and HWR pulse-trains were generated from mean spike rates averaged across the offset recording window (Figure 13). ITD sensitivity was assessed using a Poisson regression model of up to 4^th^ order. Of the units that had a significant off response (112 units), more units were ITD sensitive in response to HWR (26 units = 23.2%), compared with SAM pulse trains (22 units = 19.6%).

**Figure 14:**
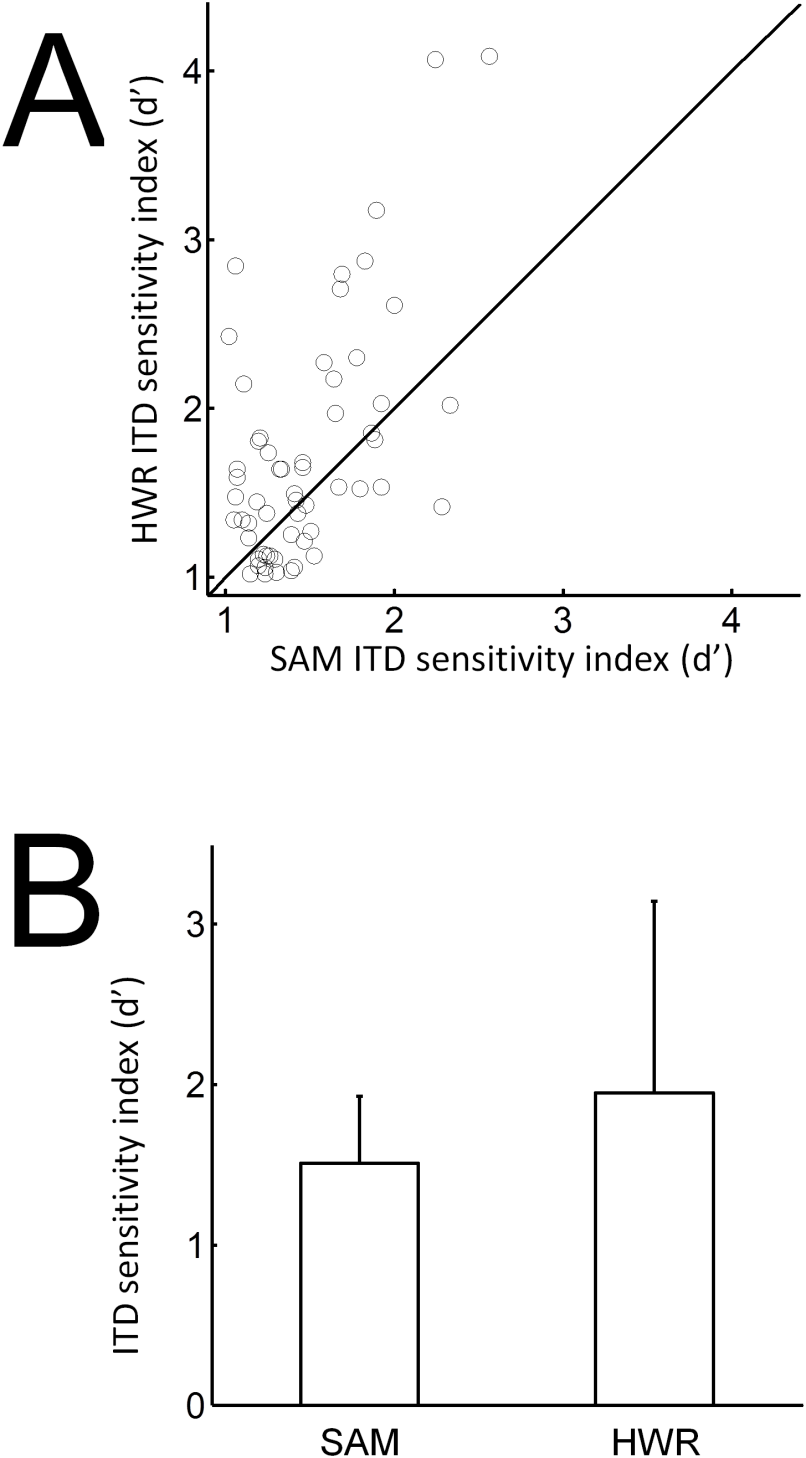
ITD sensitivity index in deaf animals with cochlear implants. (A) ITD sensitivity index for individual units plotted for offset responses to SAM and HWR pulse trains. (B) Mean ITD sensitivity index (± SD) plotted against stimulus condition for offset responses.

Figure 14 plots the ITD sensitivity index for off responses. A paired t-test revealed that the ITD sensitivity index was significantly higher in response to HWR, compared with SAM pulse-trains (P = 0.002). Again, the mean ITD sensitivity index did not vary significantly over the narrow range of modulation frequencies tested.

## Discussion

This paper demonstrates for the first time that envelope enhancement can increase neural sensitivity to ITDs in ferrets with bilateral CIs. Without envelope enhancement i) fewer cortical neurons were sensitive to ITDs and ii) those that did respond demonstrated weaker binaural interactions. Consistent with recordings from guinea-pig IC [16], our data also suggests that sharpening the acoustic envelope of sounds improves ITD sensitivity within the auditory cortex of normal-hearing animals. Any direct comparison between acoustic and electrical stimulation is problematic due to i) differences in dynamic range, ii) relative experience with electric and acoustic hearing and, iii) specific to our electric-hearing dataset, the constraint of recording cortical offset responses alone. With these caveats in mind, neural sensitivity to envelope ITDs in deaf animals with bilateral CIs appeared similar to that seen in normal-hearing animals.

Likewise, animal electrophysiology and human psychophysics should be compared with caution due to species differences, anesthesia and questions concerning the contribution of single cortical neurons to behavioral responses [30]. However, our data are broadly consistent with psychophysical studies in normally-hearing humans [5], [6], [7], [8], [9] and in deaf participants with bilateral cochlear implants [8] to suggest that ITD sensitivity crucially depends upon envelope enhancement. Together these data suggest that bilateral cochlear implant processing strategies may better convey interaural timing cues through enhancement of the modulation envelope.

### Comparison with previous animal electrophysiology

Envelope ITD sensitivity of IC neurons has been previously demonstrated in deaf cats [25]. Specifically, Smith and Delgutte [25] found, across a range of modulation frequencies, that envelope ITD sensitivity to SAM pulse trains in deaf animals is generally similar to that seen in normal-hearing animals using SAM tones. Furthermore, in IC of acutely-deafened cats [25], [31] and auditory cortex of cats with congenital hearing loss [32], widespread neural sensitivity to fine structure ITDs has been demonstrated using unmodulated pulses. Whereas unmodulated pulses have an infinite slope, a sinusoidal modulating envelope has a slower rise time. Subsequently, Smith and Delgutte [25] speculated that the slope of the amplitude envelope could enhance neural sensitivity to ITDs. Although this hypothesis is consistent with our analysis of cortical offset responses, onset recordings were complicated by electrical artifact. Given that envelope enhancement increased binaural interactions in both onset and offset responses to acoustic stimuli, it would seem reasonable to speculate that electrical stimulation with envelope enhancement would increase ITD sensitivity in onset as well as offset responses.

Although the present study is the first to investigate the effects of envelope shape on neural sensitivity to interaural envelope delays with bilateral CIs, neural sensitivity to ITDs in the envelope and fine structure of an acoustic signal has previously been demonstrated at various levels of the auditory pathway, including the medial superior olive (MSO) [33], [34], lateral superior olive (LSO) [20], [35], [36], IC [16], [37], [38], [39] and auditory cortex [28], [32], [40], [41], [42], [43]. Experience from our own laboratory, suggests that cortical neurons exhibiting binaural sensitivity in both onset and offset responses are common [28]. Psychophysical evidence suggests that sound offsets serve as an important cue in the acoustic startle reflex [44], perception of sound duration [45], consonant identification [46] and perceptual grouping [47]. Binaural offset cues also contribute to spatial localization and motion detection [48] and it has been speculated that they may even contribute to the fine-tuning of non-auditory processes, such as head-turns or arm movements [28].

Whilst it has been shown that cortical response properties differ between awake and anaesthetized preparations [49], binaural responses are commonly seen in auditory cortex under anesthesia [28]. Since the effects of anesthesia on cortical activity are unlikely to depend on the envelope shape of the stimulus, it seems reasonable to hypothesize that envelope enhancement would increase ITD sensitivity in awake as well as anaesthetized animals.

### Mechanisms of increased sensitivity to envelope enhancement

Sensitivity to ITDs is generally worse for high-, compared with low-frequency stimuli [2], [3]. This poor sensitivity to ITDs at higher frequencies may be partly explained by peripheral mechanisms [50]. Specifically, models of auditory nerve firing patterns suggest that low-frequency tones produce distinct “off periods”, where the firing probability is zero. Although modeled responses to SAM tones produce firing patterns with temporal information, these patterns lack off periods. Interestingly, modeled firing patterns to HWR envelopes include off periods similar to those associated with a low-frequency tone [5], [6], [16]. Therefore it has been suggested that the increase in ITD sensitivity observed with HWR stimuli may result from release from adaptation effects due to the longer off periods in high-frequency auditory nerve fibers [5]. Furthermore, evidence from auditory nerve recordings in cats [51] and IC recordings in guinea pigs [16] suggests that phase locking is enhanced in response to HWR compared to SAM envelopes.

The acoustic spectra of SAM and HWR tones have most energy at the tonal carrier frequency (*f_c_*) and decrease at frequencies to either side of that maximum [5]. Whilst these spectral side bands depend on the modulation frequency (*f_m_*) of the SAM or HWR stimulus, compared with SAM tones, HWR tones have additional side-band components. Specifically, SAM tones are characterized by three frequency components: the carrier frequency (*f_c_*) and two spectral side-bands (*f_c_*+*f_m_* and *f_c_*−*f_m_*). HWR tones have additional side bands spaced at multiples of twice the *f_m_*. In psychophysical studies it is necessary to limit the spectra of HWR tones to prevent the use of additional frequency components that would fall outside the effective filter width of auditory nerve fibers [5]. However, this effect is unlikely to contribute to enhanced ITD sensitivity in cortical neurons since the neurons themselves are tuned to a specific frequency. Therefore units are unlikely to be sensitive to spectral cues at frequencies away from the best frequency [16].

### Comparison with human psychophysics

The data from auditory cortex of ferrets presented here is consistent with studies that showed enhanced ITD sensitivity using trapezoid carriers with longer offset times in humans with bilateral CIs and normal-hearing listeners [8]. The offset time is the interval that the envelope remains at the minimum amplitude in each period of the stimulus. Laback and colleagues [8] showed that increasing the offset time, envelope slope and peak level of a trapezoid carrier improved ITD sensitivity to acoustic stimuli. They suggested that longer offset times may allow neurons to better recover and thus to respond more accurately to the following envelope rise [39].

PSTHs associated with HWR and SAM stimuli were broadly similar (Figs. 6 and 12 respectively under acoustic and electrical stimulation). In contrast, neural ITD sensitivity increased significantly in response to HWR stimulation, compared with corresponding SAM stimuli (Figs. 9 and 14). Therefore this increased sensitivity cannot be accounted for by changes in firing rate alone. This supports the hypothesis in psychophysical studies that the increased ITD sensitivity when using a trapezoidal carrier relates to better recovery of neural function between envelope rises.

Laback and colleagues [8] also found large inter-subject variability in ITD sensitivity amongst individuals with bilateral CIs. It is possible that factors such as mismatching the place of electrical stimulation across the ears and reduced neural survival could account for some of this variation. Furthermore, with the exception of the Simultaneous Analog Stimulation strategy (Advanced Bionics Corporation), most commercially-available implant strategies are based on pulsatile stimulation, which do not currently provide useful fine structure information. Any fine structure ITDs that arise between pulses in each ear depend more upon when each implant was switched on than the location of the input signal. Therefore, binaural perception with most implant strategies is presumed to be listener specific and may partly depend upon a listener’s ability to utilize envelope ITD cues, whilst ignoring uninformative fine structure information.

### Potential benefit of envelope enhancement to CI processing strategies

Despite limitations in the temporal coding of neurons to high frequency sounds, the results presented here suggest sensitivity of cortical neurons to ITDs increases with envelope enhancement. These findings are relevant to individuals with bilateral cochlear implants because most current commercially-available devices partially restore ILDs and envelope ITDs alone. Envelope enhancement could be used in a CI processing strategy to improve binaural perception. Laback and colleagues [8] suggested that this could be achieved by modifying the acoustic-to-electric amplitude-mapping function or by means of an algorithm to reduce the amplitude of the input signal in short temporal segments. Furthermore, Green and colleagues [52] have already used monaural envelope enhancement at the fundamental frequency to encode information about voice pitch. Although envelope enhancement algorithms could be developed for bilateral use, it would be important to ensure that any benefits to binaural hearing outweigh any potential detrimental effects on speech perception. It is also possible that the benefit of envelope enhancement on binaural perception could be further enhanced with a coding strategy that is designed to improve binaural fine structure information [10].
